# Supplementary material for: An ER translocon for multi-pass membrane protein biogenesis
Source: eLife. 2020 Aug 21;9:e56889. doi: 10.7554/eLife.56889 (PMC7505659; doi:10.7554/eLife.56889)
Supplement: Transparent reporting form [file elife-56889-transrepform.pdf]

eLIFE
1st Floor
24 Hills Road
Cambridge CB2 1JP, UK
P 01223 855340
W elifesciences.org
T @elife

eLife's transparent reporting form

We encourage authors to provide detailed information *within their submission* to facilitate the interpretation and replication of experiments. Authors can upload supporting documentation to indicate the use of appropriate reporting guidelines for health-related research (see EQUATOR Network), life science research (see the BioSharing Information Resource), or the ARRIVE guidelines for reporting work involving animal research. Where applicable, authors should refer to any relevant reporting standards documents in this form.

If you have any questions, please consult our Journal Policies and/or contact us: editorial@elifesciences.org.

Sample-size estimation

- You should state whether an appropriate sample size was computed when the study was being designed
- You should state the statistical method of sample size computation and any required assumptions
- If no explicit power analysis was used, you should describe how you decided what sample (replicate) size (number) to use

Please outline where this information can be found within the submission (e.g., sections or figure legends), or explain why this information doesn't apply to your submission:

Sample size computation and power analysis were not performed.

Replicates

- You should report how often each experiment was performed
- You should include a definition of biological versus technical replication
- The data obtained should be provided and sufficient information should be provided to indicate the number of independent biological and/or technical replicates
- If you encountered any outliers, you should describe how these were handled
- Criteria for exclusion/inclusion of data should be clearly stated
- High-throughput sequence data should be uploaded before submission, with a private link for reviewers provided (these are available from both GEO and ArrayExpress)

Please outline where this information can be found within the submission (e.g., sections or figure legends), or explain why this information doesn't apply to your submission:

eLife Sciences Publications, Ltd is a limited liability non-profit non-stock corporation incorporated in the State of Delaware, USA, with company number 5030732, and is registered in the UK with company number FC030576 and branch number BR015634 at the address 1st Floor, 24 Hills Road, Cambridge CB2 1JP | August 2014

Affinity purification of 3xFlag-TMCO1-ribosome complexes was robust and reproducible, as evidenced by data presented in different experiments throughout the manuscript.

The interaction analysis in Fig 1 is from a single mass spectrometry experiment using samples processed in parallel (membranes from wild-type vs. 3xFlag-TMCO1 cells). Only proteins with two or more unique peptide matches were considered confidently identified. Strongest hits were confirmed by western blot in multiple different experiments.

Cross-linking mass spectrometry in Fig 2-figure supplement 1 is from a single experiment. The search database included 82 human ribosomal protein subunits and all eight of the membrane associated components, which were confirmed to be the dominant components of the sample by mass spectrometry analysis of late eluting (non-crosslinked) SEC fractions.

Poor cryoEM images were excluded by manual inspection of Thon ring quality. Particles lacking density for the translocon were identified by classification and excluded.

The mRNA seq experiments in Fig 5 are from three biological replicates (cells were separately grown, processed and sequenced for each condition).

Steady-state EAAT1 protein expression levels in different genetic backgrounds (Fig 5E-G) were quantified in western blots from multiple independent biological replicates (n=12 in TMCO1 KO cells, n=3 in TRAM KO cells, and n=4 in all other KO cells).

EAAT1 mRNA levels were quantified by qRT PCR in biological duplicates (n=2) (Fig 5—figure supplement 1C).

Statistical reporting

- Statistical analysis methods should be described and justified
- Raw data should be presented in figures whenever informative to do so (typically when N per group is less than 10)
- For each experiment, you should identify the statistical tests used, exact values of N, definitions of center, methods of multiple test correction, and dispersion and precision measures (e.g., mean, median, SD, SEM, confidence intervals; and, for the major substantive results, a measure of effect size (e.g., Pearson's r, Cohen's d)
- Report exact p-values wherever possible alongside the summary statistics and 95% confidence intervals. These should be reported for all key questions and not only when the p-value is less than 0.05.

Please outline where this information can be found within the submission (e.g., sections or figure legends), or explain why this information doesn't apply to your submission:

Individual data points from replicates are shown in Figure 5G (EAAT1 protein levels) and Figure 5-figure supplement 1C (qRT PCR analysis of EAAT1 mRNA levels), along with mean and standard deviation.

(For large datasets, or papers with a very large number of statistical tests, you may upload a single table file with tests, Ns, etc., with reference to sections in the manuscript.)

Group allocation

- Indicate how samples were allocated into experimental groups (in the case of clinical studies, please specify allocation to treatment method); if randomization was used, please also state if restricted randomization was applied
- Indicate if masking was used during group allocation, data collection and/or data analysis

Please outline where this information can be found within the submission (e.g., sections or figure legends), or explain why this information doesn't apply to your submission:

No grouping, randomization or masking was used in this work.

Additional data files ("source data")

- We encourage you to upload relevant additional data files, such as numerical data that are represented as a graph in a figure, or as a summary table
- Where provided, these should be in the most useful format, and they can be uploaded as "Source data" files linked to a main figure or table
- Include model definition files including the full list of parameters used
- Include code used for data analysis (e.g., R, MatLab)
- Avoid stating that data files are "available upon request"

Please indicate the figures or tables for which source data files have been provided:

Annotated spectra corresponding to the reported ribosome-translocon cross-links are available at the MS-Viewer website (http://msviewer.ucsf.edu/prospector/cgi-bin/msform.cgi?form=msviewer) with the following accession keys: HCD data: 7s2yb4zfjw and ETD data: vdibnsypj7. Cryo-EM maps have been deposited in the Electron Microscopy Data Bank with accession codes: EMD-21426 (Map 1), EMD-21427 (Map 2) and EMD-21435 (Map 3). Coordinates for the human 60S-translocon complex have been deposited in the Protein Data Bank with accession code 6W6L. mRNA sequencing data have been deposited in Gene Expression Omnibus (GEO) under accession number GSE134027.
